# Association Between Neck Circumference and Coronary Heart Disease: A Meta-analysis

**DOI:** 10.31372/20190401.1031

**Published:** 2019

**Authors:** Guang-Ran Yang, Timothy D. Dye, Martin S. Zand, Thomas T. Fogg, Shen-Yuan Yuan, Jin-Kui Yang, Dongmei Li

**Affiliations:** a Department of Endocrinology, Beijing Tongren Hospital, Capital Medical University, Beijing, China; b Clinical and Translational Science Institute, School of Medicine and Dentistry, University of Rochester, Rochester, NY, USA

**Keywords:** neck circumference, coronary heart disease, cardiovascular disease

## Abstract

*Aims*: Neck circumference (NC) was found to be related to the risk factors for coronary heart disease (CHD). However, the effects of NC on CHD are still controversial. To evaluate the relationship between NC and CHD, a meta-analysis of observational studies was performed.

*Method*: Eligible studies on the association between NC and CHD were searched in Medline, Embase, Ovid, and Web of Science databases published in English from January 1980 to December 2016. Moreover, studies published in Chinese in Wanfang and China Hospital Knowledge databases were also searched. Random effects models in the metafor package in statistical analysis software R 3.3.3 were used for the meta-analysis. Heterogeneity was analyzed with *Q* statistics.

*Results*: Eight studies were selected for the meta-analysis. A larger NC was associated with a higher prevalence of CHD (OR = 1.18, 95% CI 1.04–1.34, *p* = 0.0108). The eight studies were further divided into three subgroups according to the criteria for diagnosing CHD. In the subgroup of coronary angiography, NC was also found to be associated with the prevalence of CHD with low heterogeneity (OR = 1.17, 95% CI 1.07–1.28, *p* = 0.0007, *I*^2^ = 17.02%). However, in the subgroup of computed tomography or past history, no association between NC and CHD was found. In addition, subgroup analyses were also conducted according to the regions of the study. No association between NC and CHD was identified in either Chinese studies or Brazil studies (OR = 1.20, 95% CI 0.96–1.49; OR = 1.31, 95% CI 0.82–2.09, respectively).

*Conclusion*: Larger NC is associated with increased risk of CHD, especially when coronary angiography was taken to diagnose CHD.

## Introduction

Coronary heart disease (CHD) is one of the most common chronic non-communicable diseases in the world. CHD is also the leading reason of all-cause deaths in adults in many countries, accounting for 30.8%–40% of all deaths worldwide (Mozaffarian et al., 2016; National Center for Cardiovascular Disease, 2012). Identifying more risk factors associated with CHD is important for CHD prevention and management.

In recent decades, the prevalence of CHD increased dramatically, and the onset age of CHD significantly decreased with more CHD patients younger than 40 years old. Obesity has been shown to be an important risk factor for CHD, and always accompanied by multiple metabolic abnormalities, such as insulin resistance, diabetes, hypertension, dyslipidemia, and gout. Obesity now is a global health problem, not only in the general population (Finucane et al., 2011), but also in islanders (Finucane et al., 2011; Okihiro & Harrigan, 2005). Several anthropometric indexes, such as body mass index (BMI), waist circumference, waist-to-hip ratio, and neck circumference (NC), are used to evaluate obesity. Several studies have found that upper-body obesity had a stronger association with insulin resistance, diabetes, dyslipidemia, and gout than lower-body obesity (Kissebah et al., 1982; Laakso, Matilainen, & Keinanen-Kiukaanniemi, 2002). NC, as an index for upper-body subcutaneous adipose tissue distribution, has been evaluated in relation to cardiovascular risk factors (Sjostrom, Hakangard, Lissner, & Sjostrom, 1995). The association between NC and insulin resistance (Laakso et al., 2002; Liang et al., 2013; Yang, Samarasinghe, Kane, Amiel, & Aylwin, 2010) and biochemical components of metabolic syndrome (MS) has also been studied (Ben-Noun & Laor, 2006; Kumar, Ismail, Mahesha, Girish, & Tripathy, 2014; Onat et al., 2009). NC has been found to be an independent predictive contributor to cardio-metabolic syndrome (Zhou et al., 2013) and early stage atherosclerosis (Liang et al., 2014).

However, the effects of NC on predicting CHD and CHD events are still controversial (Arjmand, Shidfar, Nojoomi, & Amirfarhangi, 2015; Dai, Wan, Li, & Jin, 2016; Preis et al., 2010). Preis et al. (2010) reported that in the Framingham Heart Study, NC was associated with CHD risk factors even after adjustment for visceral adipose tissue and BMI. However, in a secondary analysis using incident of cardiovascular disease as an outcome, there was no statistically significant association observed between NC and cardiovascular disease in multivariable-adjusted models. In a prospective cohort study performed in China on 12,151 high-risk cardiology outpatients from 2004 until 2014, it was found that a higher NC indicated a higher incidence of future fatal and non-fatal CHD events and all-cause mortality in both male and female high-risk population (Dai et al., 2016). A cross-sectional study performed on people who underwent coronary angiography showed that NC was a better predictor of the risk of coronary artery disease compared to other anthropometric indices (Arjmand et al., 2015).

The controversial results from different studies on association between NC and CHD make it necessary to perform a meta-analysis to evaluate the relationship between NC and CHD by combining the data of all relevant studies. The meta-analysis follows the guideline provided in the Meta-analysis Of Observational Studies in Epidemiology (MOOSE) (Stroup et al., 2000).

## Methods

### Eligibility Criteria

**Figure 1:**
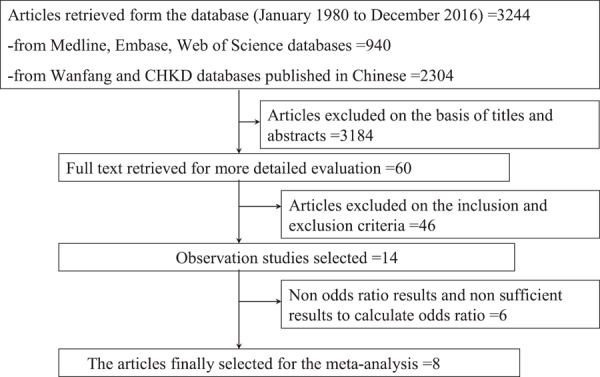
Flow diagram of article selection. *Abbreviations*: CHKD: China Hospital Knowledge Database.

Eligible studies on the association between NC and CHD were searched in Medline, Embase, Ovid, and Web of Science databases published in English from January 1980 to December 2016. Moreover, studies published in Chinese from January 1980 to December 2016 in Wanfang and China Hospital Knowledge Database (CHKD) databases were also searched. The key words “neck circumference,” “cardiovascular disease,” “coronary heart disease,” and combinations of these were used. References cited in the retrieved articles were also examined to find relevant studies that had not been identified by database searches. The articles were first selected through title and abstract screening. The secondary abstract review was performed on the first screened articles with review of the full text. The final inclusion of articles was determined by consensus between two co-authors. Figure 1 shows the search strategy in the meta-analysis.

### Selection Criteria

Inclusion criteria included: (i) observation or cohort studies; (ii) adult patients aged 18 years or older; (iii) articles that reported the association between NC and CHD in terms of odds ratios (ORs) or correlation coefficients (*r*) or other forms of effect sizes. We excluded: (i) abstracts, letters, editorials, expert opinions, case reports, and reviews; (ii) studies that included people aged less than 18 years. Disagreement was solved by the discussion within the team authors.

### Data Extraction

Data were extracted using standardized forms. Data recorded included the first authors’ names, years of publication, the locations of studies, study design, numbers of total patients, and adjusted ORs with corresponding 95% confidence intervals (CIs).

### Statistical Analysis

Random effects models were utilized to obtain the summary ORs and their 95% CIs for the association between NC and CHD in all eligible studies and subgroup analysis. Statistical heterogeneity among studies was evaluated using the Cochran *Q* statistics. The heterogeneity was further quantified by inconsistency index (*I*^2^) with 25%, 50%, and 75% representing the evidence of low, moderate, and high heterogeneity, respectively (Higgins & Thompson, 2002; Higgins, Thompson, Deeks, & Altman, 2003). According to the *p* values <0.10 of the heterogeneity test, random effects model were used to estimate OR and corresponding 95% CI. Studies using correlation coefficient (*r*) as effect sizes were converted to log odds ratios for the meta-analysis.

The funnel plot and radial plot were generated to assess the publication bias. Egger regression asymmetry test for funnel plot asymmetry was also performed to detect publication bias. The metafor package in statistical analysis software R was used for the meta-analysis (Viechtbauer, 2010), and *p*-values less than 0.05 were considered statistically significant for testing association between NC and CHD.

### Sensitivity and Subgroup Analyses

Sensitivity analyses were conducted to measure the robustness of the result. The study with the biggest contribution was removed from the meta-analysis. Then, the two studies using correlation coefficients as effect sizes were removed. Subgroup analysis was based on different nations and different diagnosis criteria for CHD were performed.

## Results

### Search Results

The screening process for articles used for the meta-analysis is shown in Figure 1. A search of PubMed, Web of Science, and Embase identified a total of 940 articles, and a search of Wanfang and CHKD database identified 2,304 articles. After title and abstract review, 60 studies were selected for a more detailed assessment. We filtered out a total of 46 studies that did not meet the criteria. Finally, eight studies were selected for meta-analysis.

A total of eight articles were included for the meta-analysis. Of them, three were conducted in China (Dai et al., 2016; Liao, Liu, Wang, & Zhang, 2015; G. Yang et al., 2016), three in Brazil (Baena et al., 2016; Chagas et al., 2011; Zen et al., 2012), one in the United States (Pokharel et al., 2014), and one in Iran (Arjmand et al., 2015). In terms of study design, there were seven cross-sectional studies and one cohort study. The sample size varied from 300 (Arjmand et al., 2015) to 12,515 (Dai et al., 2016) (Table 1).

**Table 1:** Characteristics of Studies Included in the Meta-analysis

First author (Publication year)	Location	Study design	Total number (*n*)	Control number (*n*)	CHD number (*n*)	Odds ratio	95% confidence intervals
Zen et al. (2012)	Brazil	Case-control study	376	221	155	2.40	1.10–5.30
G. Yang et al. (2016)	China	Cross-sectional study	3,176	2,560	616	1.022	0.996–1.048
Liao et al. (2015)	China	Case-control study	677	310	367	1.128	1.075–1.185
Arjmand et al. (2015)	Iran	Cross-sectional study	300	68	231	1.207	1.004–1.451
Pokharel et al. (2014)	United States	Cross-sectional study	845	323	522	1.11	0.94–1.31
Baena et al. (2016)	Brazil	Cross-sectional study	3,929	3,004	1,148	0.94	0.78–1.13
Dai et al. (2016)	China	Cohort study	12,515	7,871	2,304	1.49^a^	1.40–1.59^a^
Chagas et al. (2011)	Brazil	Cross-sectional study	337			1.39^a^	0.96–2.01^a^

CHD: coronary heart disease.

^a^ Calculated according to the data information in the original studies.

### Meta-analysis

**Figure 2:**
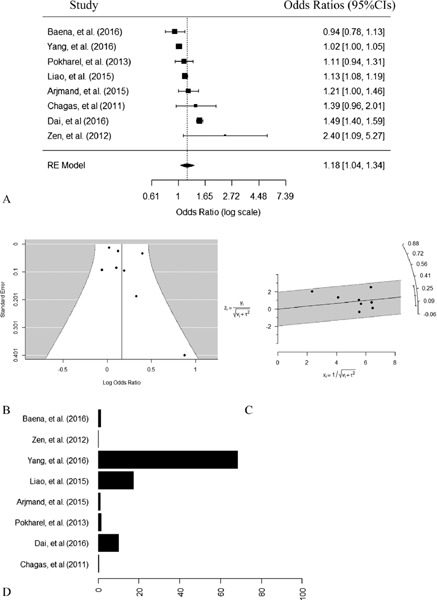
Meta-analysis of neck circumference associated with coronary heart disease (random effects model). (A) Forest plot. (B) Funnel plot. The *X*-axis was log odds ratio, and the *Y*-axis represents the standard error. No publication bias was found using regression test for funnel plot asymmetry (*p* = 0.1574). (C) Radial plot. *x* stands for the inverse of the standard errors, *y* stands for the outcome, *z* stands for standardized outcome, both *v* and τ stand for standard errors with *v* denoting the standard errors from the fixed effects part and τ denoting the standard errors from the random effects part. (D) Contribution plot.

The random-effects model was selected for data analysis, as the test for heterogeneity showed a statistically significant result (*I*^2^ = 93.06%, *p* < 0.05). The weighted odds ratio estimated from the random-effects model indicated that a higher NC was associated with a higher prevalence of CHD (OR = 1.18, 95% CI 1.04–1.34, *p* = 0.0108), as shown in Figure 2.

### Publication Bias

The homogeneity was analyzed with *Q* statistics and *I*^2^. Heterogeneity was found to be statistically significant (*Q* = 132.2043, *I*^2^ = 94.05%, *p* < 0.05). The publication bias was assessed using funnel plot and radial plot because the heterogeneity existed. The funnel plot showed relative symmetry (Figure 2). Egger regression test for funnel plot asymmetry was performed, indicating no significant bias among these studies (*z* = 1.4140, *p* = 0.1574).

### Sensitivity Analysis

**Figure 3:**
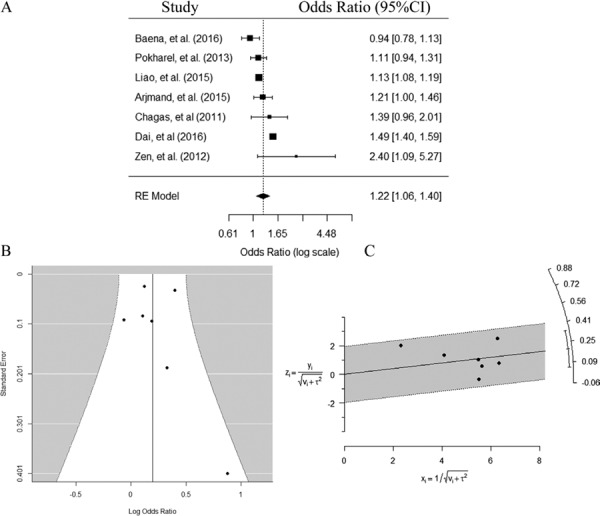
Meta-analysis of neck circumference associated with coronary heart disease after removing the study with the biggest contribution (random effects model). (A) Forest plot. (B) Funnel plot. The *X*-axis was log odds ratio, and the *Y*-axis represents the standard error. No publication bias was found using regression test for funnel plot asymmetry (*p* = 0.2466). (C) Radial plot. *x* stands for the inverse of the standard errors, *y* stands for the outcome, *z* stands for standardized outcome, both *v* and τ stand for standard errors with *v* denoting the standard errors from the fixed effects part and τ denoting the standard errors from the random effects part.

To measure the robustness of the result, sensitivity analyses were conducted. According to the contribution plots of each study, the study with the largest contribution was done by G. Yang et al. (2016), as shown in Figure 2. First, we removed the study with the largest contribution and analysed the remaining results. NC remained to be associated with CHD in a random-effects model (OR = 1.22, 95% CI 1.06–1.40, *p* = 0.0063, Figure 3). The *I*^2^ for heterogeneity was 87.78% (*p* < 0.05). The funnel plot showed relative symmetry (Figure 3). Egger regression test for funnel plot asymmetry model indicated no significant bias among these studies (*z* = 1.1586, *p* = 0.2466).

**Figure 4:**
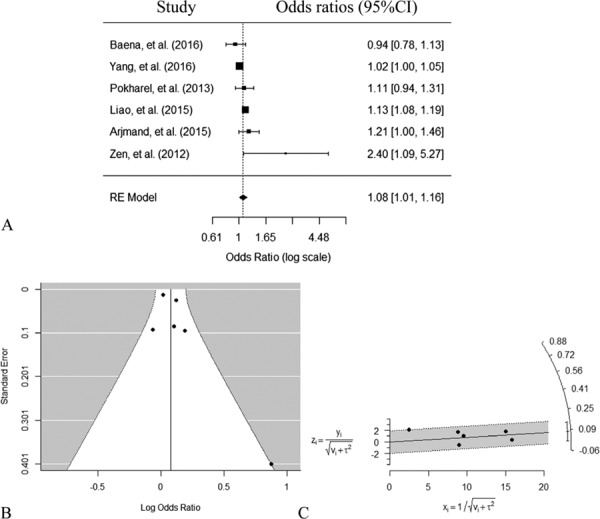
Meta-analysis of neck circumference associated with coronary heart disease after removing two studies using correlation coefficients as effect sizes (random effects model). (A) Forest plot. (B) Funnel plot. The *X*-axis was log odds ratio, and the *Y*-axis represents the standard error. No publication bias was found using regression test for funnel plot asymmetry (*p* = 0.7811). (C) Radial plot. *x* stands for the inverse of the standard errors, *y* stands for the outcome, *z* stands for standardized outcome, both *v* and τ stand for standard errors with *v* denoting the standard errors from the fixed effects part and τ denoting the standard errors from the random effects part.

Next, we removed the two studies using correlation coefficients as effect sizes (Chagas et al., 2011; Dai et al., 2016). After removing, NC was associated with CHD in a random-effects model (OR = 1.08, 95% CI 1.01–1.16, *p* = 0.0345, Figure 4). The *I*^2^ for heterogeneity was 71.09% (*p* < 0.05). The funnel plot remained relative symmetry (Figure 4). Egger regression test for funnel plot asymmetry model show no significant bias among these studies (*z* = 1.5618, *p* = 0.1183).

### Subgroup Analysis

#### Subgroup based on Different Nations

**Figure 5:**
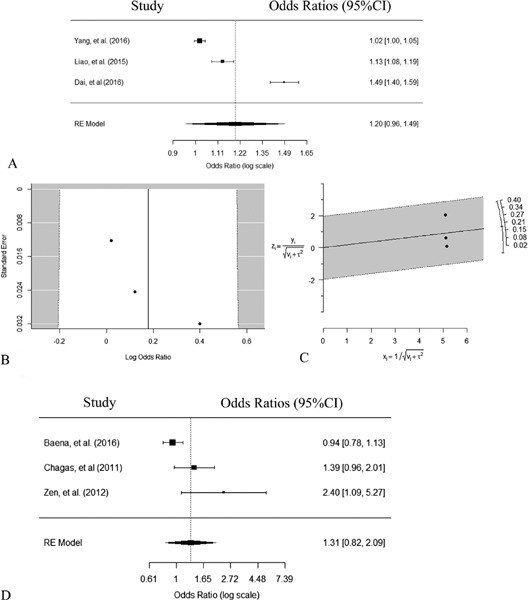
Meta-analysis of neck circumference associated with coronary heart disease in subgroups based on different nations (random effects model). (A) Forest plot for the studies conducted in China. (B) Funnel plot for the studies conducted in China. The *X*-axis was log odds ratio, and the *Y*-axis represents the standard error. No publication bias was found using regression test for funnel plot asymmetry (*p* = 0.0154). (C) Radial plot for the studies conducted in China. *x* stands for the inverse of the standard errors, *y* stands for the outcome, *z* stands for standardized outcome, both *v* and τ stand for standard errors with *v* denoting the standard errors from the fixed effects part and τ denoting the standard errors from the random effects part. (D) Forest plot for the studies conducted in Brazil.

Subgroup analysis was conducted stratified by nations. Among the eight studies in the meta-analysis, three studies were conducted in China (Dai et al., 2016; Liao et al., 2015; G. Yang et al., 2016). A random-effects model on the three studies showed no significant association between NC and CHD (OR = 1.20, 95% CI 0.96–1.49, *p* = 0.113, Figure 5). The *I*^2^ for heterogeneity was 98.7% (*p* = 0.001). The funnel plot remained relative symmetry (Figure 5). Egger regression test for funnel plot asymmetry model showed no significant bias among these studies (*z* = 2.4227, *p* = 0.0154).

There were three studies conducted in Brazil (Baena et al., 2016; Chagas et al., 2011; Zen et al., 2012). NC was not associated with CHD in a random-effects model including the three Brazil studies (OR = 1.31, 95% CI 0.82–2.09, *p* = 0.2604, Figure 5). The *I*^2^ for heterogeneity was 77.11% (*p* = 0.0193). Egger regression test for funnel plot asymmetry model show that there were no significant bias among these studies (*z* = 2.7787, *p* = 0.0055).

### Subgroup based on Different Diagnosis Criteria for CHD

**Figure 6:**
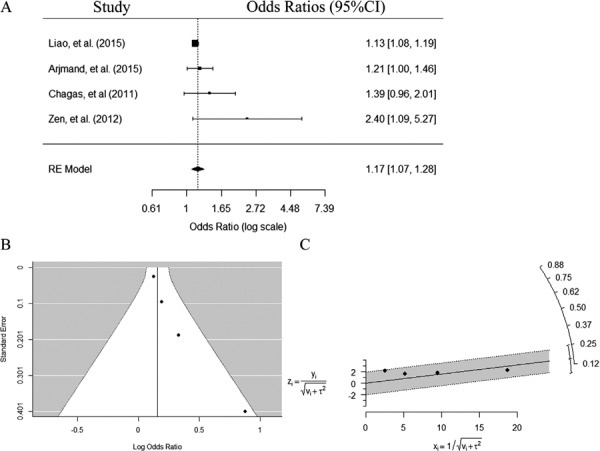
Meta-analysis of neck circumference associated with coronary heart disease in subgroup using coronary angiography to diagnose coronary heart disease (random effects model). (A) Forest plot. (B) Funnel plot. The *X*-axis was log odds ratio, and the *Y*-axis represents the standard error. No publication bias was found using regression test for funnel plot asymmetry (*p* = 0.0308). (C) Radial plot. *x* stands for the inverse of the standard errors, *y* stands for the outcome, *z* stands for standardized outcome, both *v* and τ stand for standard errors with *v* denoting the standard errors from the fixed effects part and τ denoting the standard errors from the random effects part.

There were four studies in which coronary angiography was performed to diagnose CHD (Arjmand et al., 2015; Chagas et al., 2011; Liao et al., 2015; Zen et al., 2012). NC was found to be associated with CHD in a random-effects model (OR = 1.17, 95% CI 1.07–1.28, *p* = 0.0007, Figure 6). The *I*^2^ for heterogeneity was 17.02% (*p* = 0.1668). The funnel plot remained relative symmetry (Figure 6). Egger regression test for funnel plot asymmetry model did not indicate significant bias among these studies (*z* = 2.1600, *p* = 0.0308).

**Figure 7:**
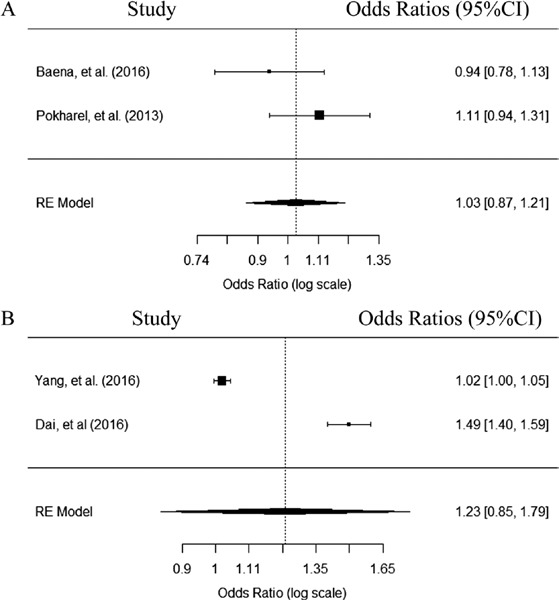
Forest plot of neck circumference associated with coronary heart disease in subgroups using computed tomography and the past history to diagnose coronary heart disease (random effects model). (A) Forest plot for studies using computed tomography to diagnose heart disease. (B) Forest plot for studies using the past history to diagnose coronary heart disease.

There were two studies in which computed tomography was performed to evaluate the conditions of the coronary arteries (Baena et al., 2016; Pokharel et al., 2014). NC was not associated with CHD in a random-effects model (OR = 1.03, 95% CI 0.87–1.21, *p* = 0.7606, Figure 7). The *I*^2^ for heterogeneity was 43.23% (*p* = 0.1844).

In the two studies, CHD was diagnosed by the history of past myocardial infarction, acute coronary syndrome, and cardiac death (Dai et al., 2016; G. Yang et al., 2016). NC was not associated with CHD (OR = 1.23, 95% CI 0.85–1.79, *p* = 0.2721, Figure 7). The *I*^2^ for heterogeneity was 99.16% (*p* < 0.001).

## Discussion

Overweight/obesity is recognized as one of the most common risk factors for CHD (Mongraw-Chaffin, Peters, Huxley, & Woodward, 2015; Wormser et al., 2011). NC was shown in many studies to be an indicator for evaluating overweight/obesity. In this meta-analysis with eight studies, NC was associated with CHD. In the subgroup analysis, when coronary angiography was performed to diagnose CHD, NC was also found to be associated with CHD with low heterogeneity. However, the association between NC and CHD was not found in the other subgroup analysis.

There are several anthropometric indicators to evaluate overweight/obesity, such as BMI, waist circumference, waist hip ratio, and NC. Several meta-analysis studies on the association between anthropometric indicators with CHD showed that BMI, measured continuously or categorically, had deleterious effects on the risk of incident CHD in people across diverse populations (Mongraw-Chaffin et al., 2015). In a meta-analysis study of 82,864 individuals from nine nationwide British cohorts, greater waist circumference and waist hip ratio were found to be associated with an increased cardiovascular mortality (Czernichow, Kengne, Stamatakis, Hamer, & Batty, 2011). However, there was a lack of meta-analysis about the association between NC and CHD.

Since NC was first evaluated in relation to cardiovascular risk factors by Sjöström et al. in 1995 (Sjostrom et al., 1995), many studies were performed to assess the effects of NC in clinical practice. First, NC was used to evaluate overweight and obesity (Ben-Noun & Laor, 2003; Onat et al., 2009; L. Yang et al., 2010). Then it was used to assess obesity related disease, such as insulin resistance, obstructive sleep apnea syndrome, and metabolic syndrome (Ben-Noun & Laor, 2006; Liang et al., 2013). The prevalence of obesity increases in most countries in recent decades. In 2009–2010, the prevalence of obesity was about 35% in U.S. adults (Flegal, Carroll, Kit, & Ogden, 2012). The increase in obesity was seen not only in North America, but also in the Asian/Pacific islanders. An analysis in Australian adults aged over 45 years found that the prevalence of obesity was nearly 30% (Buchmueller & Johar, 2015). It was reported that the mean BMI increased by above 2.0 kg/m^2^ per decade from 1980 to 2008 in the Cook and Nauru Islanders (Finucane et al., 2011). The Pacific Islander Health Study had shown that the majority of the male Pacific Islander adult was already overweight by aged 18 years (84% of Samoan and 65% of Tongan males being obese) (Panapasa, McNally, Heeringa, & Williams, 2015). The association of NC with cardiovascular disease risks was also studied in Asian/Pacific Islanders (Aoi et al., 2016; Dixon & O’Brien, 2002; Lindarto, Shierly, & Syafril, 2016). A study conducted in obese premenopausal Australian women found that NC was a significant predictive factor of hyperinsulinemia (Dixon & O’Brien, 2002). An observation study conducted in Indonesia reported that NC was related to BMI and may be useful in evaluating overweight/obesity (Lindarto et al., 2016). Another prospective cohort study conducted in a Japan community found that change in NC was related to atherosclerosis-related markers (Aoi et al., 2016). Due to the observed association of NC with cardiovascular risk factors (lipid profile, insulin resistance, metabolic syndrome, and hypertension) in many studies (Ben-Noun & Laor, 2003, 2006; Liang et al., 2013; G. Yang et al., 2010), NC might be related to CHD. However, the results of clinical studies on the association of NC with CHD remained controversial. In a cross-sectional study, NC was found to be a better predictor of the risk of CHD compared to other anthropometric indices (Arjmand et al., 2015). However, the results from ELSA-Brazil study had shown that NC was not associated with coronary atherosclerosis (Baena et al., 2016).

A variety of study designs were used for this meta-analysis, with various study population (i.e., age, sex) and uncontrolled confounding factors (past history, cardiovascular risk factors). These variations may affect the heterogeneity and the results. In some studies related to NC and obesity, it was shown that NC in males was higher than that in females, so the cutoff of NC for evaluating obesity was sex-specific (G. Yang et al., 2010). However in the eight studies used in our meta-analysis, only one study assessed the sex-specific relationship between NC and CHD (Baena et al., 2016). To assess whether the difference in nation would affect the results, subgroup analyses were conducted stratified by nations. In each nation strata, no association was found between NC and CHD.

In studies used for the meta-analysis, CHD was diagnosed by using coronary angiography in four studies (Arjmand et al., 2015; Chagas et al., 2011; Liao et al., 2015; Zen et al., 2012), by using computed tomography scanner in two studies (Baena et al., 2016; Pokharel et al., 2014), and by history of past myocardial infarction, acute coronary syndrome, and cardiac death in two studies (Dai et al., 2016; G. Yang et al., 2016). In the subgroup analysis, the eight studies were stratified into three subgroups according to the criteria for diagnosing CHD. In the subgroup of coronary angiography, NC was also found to be associated with the prevalence of CHD with low heterogeneity. However, in the other subgroups, where computed tomography or past history were used to diagnose CHD, no association between NC and CHD was found. In the subgroup of computed tomography or past history, there were only two studies included in each subgroup, which may interfere with the results of the meta-analysis. In clinical practice, when people were suspected for CHD, coronary angiography will be recommended in most cases. When needed, a 64-detector computed tomography scanner is also used to evaluate the coronary atherosclerosis. Coronary angiography is recognized as the gold standard for diagnosing CHD. Based on the results of the subgroup of coronary angiography, there is statistically significant evidence that NC is associated with CHD. Furthermore, large-scale studies are needed to confirm this relationship. Whether NC can predict CHD also need prospective studies. In a Chinese cohort study, it was found that NC was related to the incident of CHD events (Dai et al., 2016). However, in the Framingham study, though NC was related to the cardiovascular risk factor, NC was not related to the incidence of cardiovascular events (Preis et al., 2010).

Due to the nature of observational studies used in the meta-analysis, the inherent biases cannot be controlled as in the randomized controlled studies, and some confounding effects are inevitable in observational studies. In addition, the study design and subject selection for each analysis are not coherent. For example, a cross-sectional study is more likely performed in a particular area with a high incidence, and is less likely in an area with a rare incidence. Therefore, the results could have bias and are difficul in generalizing the population of interest (Jung & Lee, 2009; Stroup et al., 2000).

Additional potential limitations to this meta-analysis include: (1) The limited number of studies included in the meta-analysis could affect the association between NC and CHD, for example, no statistical significant association exist between NC and CHD when the number of studies decreased to three; (2) Different diagnose criteria for CHD was used in these eight studies in the meta-analysis; (3) Participants in some studies were patients with high risk for CHD, for example, G. Yang et al. (2016) investigated the association between NC and CHD in type 2 diabetes, and Arjmand et al. (2015) investigated people with stable angina. (4) There were studies investigating the association of NC with cardiovascular risk factors in different populations, however, only several studies investigated the association of NC with CHD. The studies included in this meta-analysis were performed in Asia and America. The association of NC with CHD risk factors was also studied in Asian/Pacific Islanders (Aoi et al., 2016; Dixon & O’Brien, 2002; Lindarto et al., 2016). However, no studies evaluating the relationship between NC and CHD in Asian/Pacific Islanders were found. Further studies in this population are needed to confirm this association in Asian/Pacific Islanders. The reason for the increasing obesity in different population may be different. However, there were common risk factors for obesity in Chinese and Asian/Pacific Islands. Modern dietary pattern was reported to be positively associated with obesity in China (Xu, Hall, Byles, & Shi, 2015). Increased intake of fats and sugars was found to be one of the main reasons for obesity in the Pacific Islanders (Snowdon & Thow, 2013). Obesity is now a global public health problem. Nutrition education is a very important preventive strategy for both Chinese and Asian/Pacific Islanders. Studies on NC, an anthropometric indicator for obesity, and obesity related disease, such as CHD, would be helpful in future prevention and treatment of obesity in Asian/Pacific Islanders.

Our results indicate that NC may be associated with CHD, especially when coronary angiography was taken to diagnose CHD. Given the high incidence of CHD, further large-scale and prospective studies may be worthwhile to confirm this relationship between NC and CHD.

## Acknowledgements

We would like to thank the anonymous reviewers for their insightful comments and suggestions that helped to improve our manuscript.

## Declaration of Conflicting Interests

The authors declared no potential conflicts of interest concerning the research, authorship, or publication of this article.

## Funding

This study is supported by Capital’s Funds for Health Improvement and Research [2016-2-2054], and the Beijing Municipal Training Foundation for Highly-qualified and Technological Talents of Health System [2014-3-013]. Dr. Li’s, Dr. Zand’s, Mr. Fogg’s, and Dr. Dye’s time is supported by the University of Rochester’s Clinical and Translational Science Award (CTSA) number UL1 TR000042 and UL1 TR002001 from the National Center for Advancing Translational Sciences of the National Institutes of Health. Dr. Zand is also supported by the National Institute of Allergy and Infectious Diseases and the National Institute of Immunology, grant numbers AI098112 and AI069351. The content is solely the responsibility of the authors and does not necessarily represent the official views of the National Institutes of Health.
